# Supplementary material for: Learning curve and surgical outcome of robotic assisted colorectal surgery with ERAS program
Source: Sci Rep. 2022 Nov 29;12:20566. doi: 10.1038/s41598-022-24665-w (PMC9709162; doi:10.1038/s41598-022-24665-w)
Supplement: Supplementary file 2 — Supplementary Information 2. [file 41598_2022_24665_MOESM2_ESM.zip › RAL Raw data-Ñ[▒K/075/OP_ñw╝╨░O▒Kñσ.pdf]

正式摘要(電子簽章：000000215080236)

W95 / W95

臺中榮民總醫院

Operation Note

[redacted]

- 手術日期：2021/04/22
- 手術醫師：VS 蔣鋒帆，CR 楊曙榮，R 陳勇先
- Anesthesia : GE
- Position : Lithotomy
- Aseptic agents : Soap , Betadine Alcohol , Betadine Aqua

Diagnosis

- Pre-operative diagnosis : Malignant tumor of rectum
- Post-operative diagnosis : Ditto.

Surgical Indication

- Tumor resection

Operation

- Robot Assisted Low Anterior Resection + loop ileostomy

Finding

- 1. ulcerative tumor, about 6 cm in size 6 cm FAV.
  2. Total meso excision was complet
  3. IMA high ligation+
  4. distal margin 3 cm, Surefrom 45 *2 for rectum
  5. air tie test neg. donut intact.
  6. loop ileostomy in RLQ

Procedure

- 1. under GE, the pt was in lithotomy position
  2. the trocar hold was made in periumbilicus open method. the other 5 hole made by punctiure
  3. identify holly plain and dissct. isolated IMA root. ligated with clips
  4. dissect between Gerota fascia and meso rectum. lysis Lt white line
  5. dissect into pelvis area. dissect posterior side of rectum down to pelvis follor levetar ani. anterior side of Denovillor fascia. Total meso excision was complet
  6. resected rectum stump with Sure Form 45 2 fires.
  7. pull the specimen outside abdomen from midline wd
  8. resect Rectum
  9. embeded envril 29 mm
  10.reset laparoscopy and complete anastomsis (side to end). air tie test neg. donut intact.
  11. loop ileostomy in RLQ
  12.he tolerate whole procedure and extubated.
- Estimated blood loss: minimum
- 器械數量: 正確
- 紗布數量: 正確

Image

- PAINT
  - abdomen

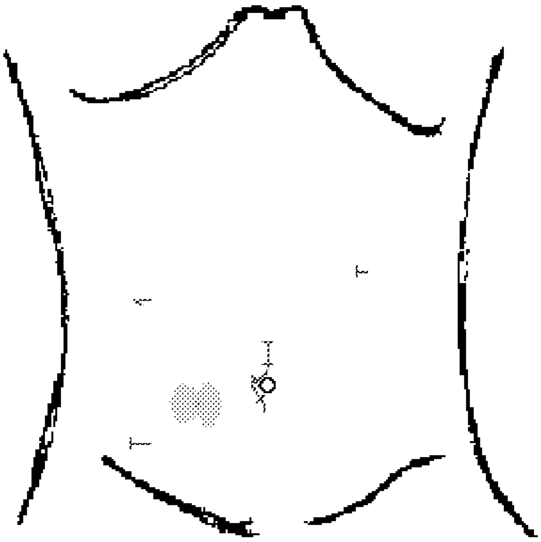

- colon & rectum

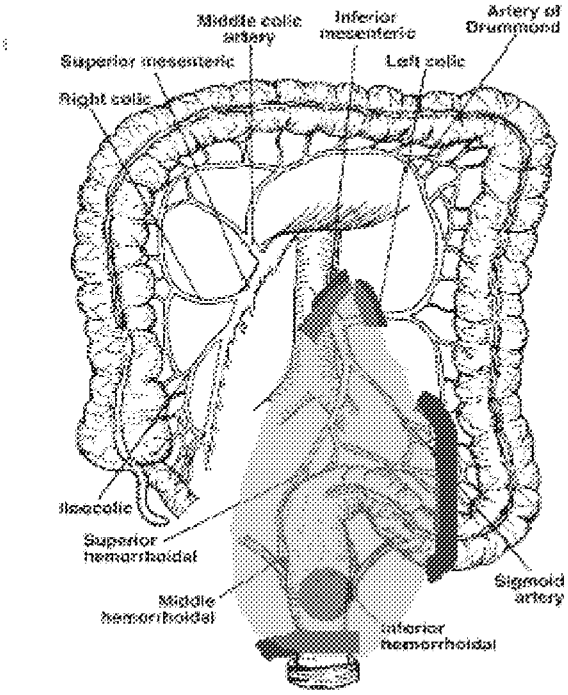

主任 2062F蔣鋒帆 / 醫師 2292G楊曙榮

正式摘要**(電子簽章：000000215080236)**

W95 / W95

doctorRank:B
